# Potential Differences in the Cardiometabolic Risk Profile of Patients with Psoriatic Disease according to Their HLA-C∗06 Status

**DOI:** 10.1155/2022/1451193

**Published:** 2022-01-27

**Authors:** Rubén Queiro, Pablo Coto-Segura, Ignacio Braña, Marina Pino, Stefanie Burger

**Affiliations:** ^1^Rheumatology Division, Hospital Universitario Central de Asturias, Oviedo, Asturias, Spain; ^2^ISPA Translational Immunology Division, Hospital Universitario Central de Asturias, Oviedo, Asturias, Spain; ^3^Oviedo University School of Medicine, Spain; ^4^Dermatology Division, Hospital Vital Alvarez Buylla, Mieres, Asturias, Spain

## Abstract

The human leukocyte antigen-C∗06 (HLA-C∗06, formerly HLA-Cw6) is the main genetic biomarker in psoriatic disease. It has been related to several phenotypic traits in psoriatic disease, but its role in relation to cardiometabolic comorbidities is unknown at present. Here, we analyze the potential connections between this biomarker and the cardiometabolic profile of these patients. We carried out a cross-sectional observational study including 400 patients recruited at a single university hospital. Clinical and classical cardiometabolic factors were compared between HLA-C∗06-positive and HLA-C∗06-negative individuals (OR with 95% CI). Multivariate regression analyses were carried out to check for disease traits associated with different cardiometabolic risk factors. The study population included 215 men (53.8%) and 185 women (46.2%), mean age of 46 ± 15 years, and an average disease evolution of 17 ± 12.6 years. Ninety-three (23.3%) patients met CASPAR criteria for psoriatic arthritis. HLA-C∗06 carriers (*n*: 160, 40%) showed an earlier age at disease onset, psoriasis family history, and more severe skin disease (type I disease). After correcting for age, sex, and disease duration, they also showed less hypertension (13.8% vs. 24.2%, OR 0.7 (95% CI: 0.42-0.78), *p* = 0.025), lower waist circumference (94.4 ± 13.7 vs. 98.3 ± 13.8 cm), and lower BMI (27 ± 4.4 vs. 28.1 ± 4.8, *p* < 0.05). We confirmed the well-known association between HLA-C∗06 and type I psoriatic disease. As a novel finding, patients carrying HLA-C∗06 showed a better cardiometabolic profile. In any case, these findings need further confirmation.

## 1. Introduction

Psoriasis is an immune-mediated skin disease affecting 2-3% of the general population. The most common companion to psoriasis is psoriatic arthritis (PsA), a chronic inflammatory arthritis that affects approximately one-third of psoriasis patients. Both are the main poles of what is currently known as psoriatic disease [1]. The underlying mechanisms of psoriatic disease appear to involve complex interactions between genetic and environmental factors leading to alterations in immunological and inflammatory pathways involving the innate and adaptive immune responses [1].

The human leukocyte antigen (HLA) class I region encodes essential proteins for immune recognition [2]. Within this region, several genes are of particular importance for susceptibility as well as for the phenotypic differentiation of psoriatic disease [2]. Thus, the HLA-C∗06 allele is the major genetic determinant for the skin component of psoriatic disease but its role on PsA susceptibility is much lower, suggesting some grade of genetic heterogeneity between both processes (joint and skin) in this condition [3].

Apart from the well-known association between HLA-C∗06 and type I psoriasis (familial, severe, early-onset), little is known about the role of HLA-C alleles on the phenotypic expression of psoriatic disease [4]. In that sense, a few studies have found an association between HLA-C∗06 and a longer psoriasis-arthritis latency time, as well as a positive association between several HLA-C antigens and different features of psoriatic disease [2, 4–6].

Although psoriatic disease is frequently accompanied by cardiovascular comorbidity [7], the potential genetic connections between this comorbidity and psoriatic disease have received little attention. Eirís et al. demonstrated that genetic variations in the IL12/23 pathway were important not only to define both the risk and severity of psoriatic disease but also for the risk of certain comorbidities such as type 2 diabetes mellitus [8]. In another study, Inerot et al. found endocrine disorders in 9% of HLA-Cw6-negative psoriasis patients compared to 1% of those Cw6 positive. In addition, no Cw6 patient was diabetic compared to a 5% overall prevalence of diabetes in the Swedish general population [9].

Furthermore, we do not know whether the genetic pathways to both cardiovascular comorbidity and psoriatic disease are common or different. However, a certain degree of commonality between psoriatic disease and some cardiovascular comorbidities as regards genes/proteins, biological processes, and inflammatory pathways has recently been highlighted [10]. Another study demonstrated shared genes between psoriasis and increased risk of dyslipidemia, hypertension, and cardiovascular disease [11]. Yet, a German study suggested that the genetic architecture of both psoriatic disease and cardiometabolic traits is largely distinct [12].

Genetic relationship studies between cardiometabolic risk factors and psoriatic disease are scarce, but they may contribute to refining the healthcare for these patients. Here, we aimed to analyze the role of the HLA-C∗06 allele on the clinical expression and cardiometabolic associations of a substantial number of patients with psoriatic disease recruited at a single university institution.

## 2. Patients and Methods

This cross-sectional observational study included 400 consecutive patients with psoriatic disease, diagnosed and followed up in a collaborative dermatology-rheumatology unit at a tertiary center in northern Spain. To study the effect of the HLA-C∗06 allele on certain characteristics of psoriatic disease, all patients with HLA-C∗06 determination who attended this unit over a six-month period were consecutively recruited for this study. This study was approved by the Ethics Committee of Clinical Investigation of Principado de Asturias (ref. HUCA 68/19), and the participants gave their written informed consent. Patient's cohort was registered as a Biobank Collection by Spanish Instituto de Salud Carlos III (reference C.0003441).

In summary, clinical, analytical, sociodemographic, radiological, and treatment variables were collected. The HLA-C∗06 allele was determined in all patients by molecular biology techniques. The main cardiometabolic risk factors, adverse cardiovascular events, lipid profile, body mass index (BMI), and waist circumference were determined. In patients with cutaneous psoriasis, the following variables were collected: type of psoriasis, its location, nail involvement, and PASI. All PsA patients met the ClASsification for Psoriatic ARthritis (CASPAR) criteria [13]. Phenotypes of peripheral arthritis were collected as follows: oligoarticular (4 or fewer affected joints) and polyarticular (5 or more). Axial involvement was defined by the Assessment of SpondyloArthritis International Society (ASAS) criteria for axial spondyloarthritis [14].

In terms of laboratory data, hemogram, blood, and urine biochemistry, immunological parameters (rheumatoid factor, antinuclear antibodies, and HLA-C∗06), and inflammatory reactants (erythrocyte sedimentation rate and C-reactive protein) were analyzed. Glucocorticoids, nonsteroidal anti-inflammatory drugs (NSAID), and conventional as well as biologic disease-modifying drug used were also collected.

Information was collected on the family history of the disease. For the purposes of this study, patients were divided into early-onset versus late-onset disease based on a cutoff age of 40 years.

### 2.1. HLA-C Profile Testing

To determine HLA-C typing, we followed the same methodology previously used by our group [6]. Briefly, DNA was isolated from lymphocytes by standard procedures. HLA-C alleles were specifically amplified with a combination of the sense primer SV1 (exon 2, codon 45) and the antisense primer SV2 (exon 3, codon 182). The spanning sequences (680 base pairs) containing the hypervariable regions of HLA-C exons 2 and 3 were used to examine the HLA-C alleles. Polymerase chain reaction conditions for the amplification of exons 2 and 3 were 95°C for 30 seconds and 67°C for 50 seconds (50 cycles), with an initial denaturation step of 98°C for 1 minute and a final extension step of 70°C for 5 minutes. The specificity of the PCR-SSOP (polymerase chain reaction sequence-specific oligonucleotide probe) method was checked by using B lymphoblastoid cell lines as positive controls for the HLA-C alleles.

### 2.2. Statistical Analysis

Central and dispersion measures were analyzed. Differences between qualitative variables were measured by a Pearson's chi-square and Fisher's exact test. To test the differences between quantitative variables, parametric (Student's *T* test) and nonparametric tests (Mann-Whitney *U* or Kruskal-Wallis *H*) were used according to the goodness of fit test. Multiple comparisons were adjusted after the Bonferroni correction. Odds ratio (OR) values with 95% CI were calculated by conditional logistic regression analysis. Initially, univariate conditional logistic regression analyses were carried out to examine unadjusted associations between the main cardiometabolic risk factors (obesity, diabetes, hypertension, and dyslipidemia) and their potential association variables. Significant variables in these univariate regression analyses (*p* < 0.10) were then introduced in multivariate analyses with a backward stepwise approach. As this was a merely descriptive study, an a priori sample size was not determined. The level of significance was set below 5%. Data were analyzed using SPSS V19.0 statistical software (IBM Corp., NY, USA).

## 3. Results

### 3.1. Summary of Study Population

The study included 215 men (53.8%) and 185 women (46.2%), with a mean age of 46 ± 15 years, a mean age of onset of psoriasis of 29 ± 17.5 years, and an average time of disease evolution of 17 ± 12.6 years.  Table 1 summarizes the main characteristics of the study population.

### 3.2. Analysis by Gender

With respect to women, men showed significant differences in serum HDL-C levels (51 ± 12.5 mg/dl vs. 60 ± 15.7 mg/dl), triglyceride levels (144 ± 86.4 mg/dl vs. 108 ± 67.8 mg/dl), abdominal circumference (102 ± 11.4 cm vs. 92 ± 14.2 cm), and BMI (28.5 ± 4.2 vs. 27.2 ± 5.1); *p* < 0.05 for all comparisons after the Bonferroni correction. Regarding the qualitative variables, more men than women presented nail psoriasis (68% vs. 54%, *p* = 0.003). However, the frequency of PsA did not differ between men (22%) and women (25%).

### 3.3. Analysis of the PsA Population

Overall, 93 (23.3%) patients met CASPAR criteria for PsA (47 men and 46 women, mean age 49 ± 12.5 years). The distribution of joint patterns was as follows: oligoarthritis (43%), polyarthritis (28%), axial (7.5%), and mixed (21.5%). Psoriasis duration was significantly higher among PsA patients (22 ± 14.5 vs. 16 ± 12.4 years, *p* < 0.05). The PASI average was significantly higher among PsA patients (14 ± 11.2 vs. 11 ± 8.2, *p* < 0.05). Consequently, more patients with PsA had PASI values greater than or equal to 10 (51.6% vs. 37.1%, OR 1.9 (95% CI: 1.5-4.2), *p* = 0.002). Moreover, more PsA patients had nail disease (72% vs. 56.4%, OR 2.4 (95% CI; 1.8-4.4), *p* < 0.001). Both diabetes (15.1% vs. 9.1%, *p* = 0.022) and dyslipidemia (19.4% vs. 13.7%, *p* = 0.08) were more prevalent in patients who developed PsA.

### 3.4. Analysis of Cardiovascular Risk Factors

Although most cardiovascular comorbid factors were significantly more prevalent in subjects with disease onset above 40 years in bivariate analyses (*p* < 0.05), after the fully adjusted multivariate models, subjects with disease onset above 40 years had a higher risk of developing diabetes (OR 2.6, CI 95%: 1.14-5.93, *p* = 0.022).

### 3.5. Analysis by HLA-C∗06 Status

Of the 400 patients in the study, 160 were HLA-C∗06 carriers (40%), 88 males and 72 females. The mean age of HLA-C∗06-positive patients was significantly lower (43.3 ± 13.8 years) than that of negative subjects for this marker (48 ± 15 years) (*p* < 0.05). In addition, the mean age at psoriasis onset was significantly lower among C∗06 individuals (22 ± 14.8 vs. 32.6 ± 17.6 years, *p* < 0.05). A similar percentage of patients with (36.6%) and without (41%) PsA presented this allelic biomarker. No difference in HLA-C∗06 distribution was detected between joint patterns. A significantly higher proportion of C∗06-positive patients (50% vs. 33.3%, OR 2.2 (95% CI: 1.82-4.21), *p* = 0.024) had a PASI greater than or equal to 10. More patients carrying HLA-C∗06 showed a family history of psoriasis (55% vs. 33.3%, OR 2.4 (95% CI: 1.91-3.87), *p* = 0.018). After correcting for age, sex, and disease duration, there were fewer hypertensive patients among HLA-C∗06-positive individuals (13.8% vs. 24.2%, OR 0.7 (95% CI: 0.42-0.78), *p* = 0.025). Furthermore, after correcting for age, sex, and disease duration, patients with this genetic marker had significantly lower waist circumference (94.4 ± 13.7 vs. 98.3 ± 13.8 cm) and lower BMI (27 ± 4.4 vs 28.1 ± 4.8) (*p* < 0.05). Furthermore, the psoriasis-arthritis gap time was significantly longer in HLA-C∗06-positive patients (21 ± 14.7 vs. 15.2 ± 13.4 years, *p* < 0.05).  Table 2 summarizes the main differences between HLA-C∗06-positive versus negative individuals.

**Figure 1:**
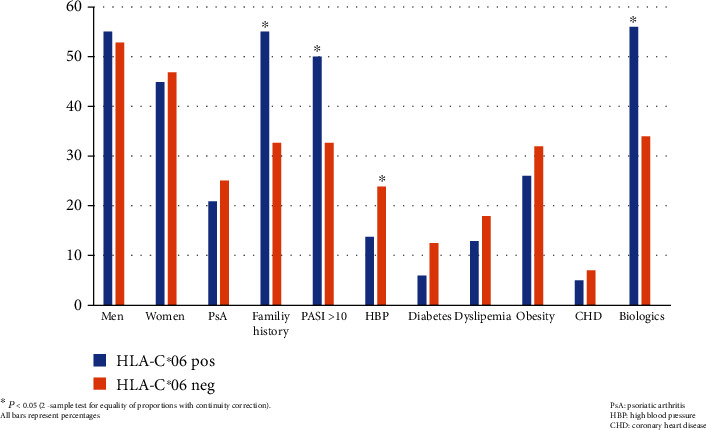
The HLA-C∗06 phenotype in psoriatic disease. Patients carrying this genetic biomarker showed more severe and widespread skin disease, a younger age at disease onset, and more family history of disease (type I disease). As a novel finding, these patients had less hypertension and lower BMI as well as lower waist perimeter, thus showing a better cardiometabolic profile. Finally, this biomarker does not seem useful to identify PsA. BMI: body mass index; PsA: psoriatic arthritis.

Figure 1 offers a summary view of the clinical phenotype associated with the HLA-C∗06 antigen in our study population.

## 4. Discussion

In this single-center study that included a substantial number of patients with psoriatic disease, we have confirmed findings from previous studies related to an association between the HLA-C∗06 allele and an earlier age of onset of psoriasis, a positive family history of the disease, and more extensive and severe skin disease (type I psoriasis) [2, 4–6]. Additionally, in line with previous studies, we confirmed that the elapsed time between the onset of psoriasis and the onset of arthritis was significantly longer in subjects carrying this genetic biomarker [3–6]. However, in our series, we were unable to confirm a different distribution of this allele between patients who developed arthritis and those who did not. This latter finding is contradictory to the findings of some studies [3, 4], although it corroborates those of others [6]. To our knowledge, our study is the first to report an inverse association between hypertension and HLA-C∗06, and between this allele and lower waist circumference as well as lower BMI.

The HLA-C∗06 allele is the main factor in the genetic etiology of psoriatic disease, being responsible for a third of this etiological burden [2, 4]. Moreover, the presence of this genetic trait has contributed to highlighting the importance of age at disease onset as a key descriptor to understand the pathogenesis, clinic, and evolution of psoriatic disease [4, 15]. All in all, most of the studies of the genetic associations between this marker and the disease refer to an association with the cutaneous domain of the disease, the connection with the musculoskeletal domain being much looser or nonexistent [2–4]. In our study, this antigen was equally distributed among the different PsA subgroups. Furthermore, the psoriasis-arthritis latency in those patients with psoriasis onset above 40 years is usually significantly shorter compared to those patients whose psoriasis debuts before that age limit, as we could demonstrate here [3, 4, 6, 15]. We also know that the etiological weight attributed to HLA-C∗06 dilutes as patients' age advances, suggesting that other etiological factors, still poorly defined, come into play from the age of 40 onwards [4, 15]. Ultimately, these findings suggest that HLA-C∗06 not only marks early-onset psoriasis but also underscores the risk of skin disease and not so much the risk of the musculoskeletal component. Another data that reinforces the connection of HLA-C∗06 with skin disease, but not with musculoskeletal disease, is that this marker is associated with extensive and severe skin disease [4], as we were also able to verify here; however, in our PsA population despite having a more severe skin disease than patients without arthritis, we did not find an overexpression of this allele.

In any case, the most striking and novel findings drawn from our study refer to the association between HLA-C∗06 and a lower prevalence of hypertension (the frequency of hypertension was reduced by 30% in individuals carrying this antigen) and the connections between this allele and a lower BMI as well as lower waist circumference. Although we detected some differences between men and women in some cardiometabolic parameters, the differences found based on the presence of HLA-C∗06 were corrected for age and sex. Globally, our study findings point out in some way to a potential link between this antigen and a healthier cardiometabolic profile. These data are interesting because they are in line with studies that find a higher prevalence of metabolic syndrome and its components, in patients with PsA [1, 7], an entity where the etiological weight of HLA-C∗06 is less or nonexistent. A potential explanation for our cardiometabolic findings could be that all traditional cardiometabolic factors tend to go in parallel with increasing age [7, 15]. The average age of HLA-C∗06-negative patients was significantly higher compared to that of HLA-C∗06-positive individuals. Therefore, it would be expected that patients carrying this antigen generally had a lower burden of cardiovascular risk factors. The prevalence of cardiovascular risk factors was higher in older subjects as would be expected. However, after correcting for age, sex, and disease duration, the association between hypertension, BMI, or abdominal circumference, and HLA-C∗06 remained significant. Indeed, the only cardiovascular risk factor associated with late disease onset was diabetes, so we hypothesize that the connection between HLA-C∗06 and lower prevalence of hypertension may be a direct relationship, although with still ill-defined mechanisms. In support of our findings, a recent study found that hypertension was more prevalent among patients with arthritis within the spectrum of psoriatic disease and in those with a higher BMI and late-onset psoriasis [16].

We have also seen how HLA-C∗06-positive patients had significantly lower BMI and reduced waist circumference (a surrogate marker for abdominal and visceral fat). Interestingly, different recently published studies show how C∗06-positive patients tend to respond better to biological therapy [17–19], while, on the contrary, overweight/obese patients tend to show worse response to, and worse persistence of, biological therapy [20, 21]. We can then hypothesize that one of the mechanisms by which patients showing C∗06-positive psoriatic disease respond better to biological therapy is related to the “protective” cardiometabolic effects linked to this biomarker. Nevertheless, the relationships between systemic treatments, both conventional and biological, and the risk of adverse cardiovascular events are complex, so that some agents (e.g., IL-17A inhibitors) seem to be associated with favorable outcomes in this regard, while in others, the opposite seems to be the case (cyclosporin A). Even with other agents, this connection seems unclear (IL12/23 inhibitors) [22].

Our study has the limitations of most observational studies with retrospective data collection. Therefore, we cannot discern the direction of the associations found in it, and the bias of false causality cannot be reliably ruled out. For example, HLA-C∗06-positive patients had a higher exposure to biological therapy, which may have biased the results of this study, although this type of exposure was considered in the backward multivariate regression model. However, being a cross-sectional study, this type of bias is not completely ruled out. On the other hand, a recent study found a positive association between HLA-C∗06 and a higher extension of subclinical atherosclerosis in carotid ultrasound [23], which forces us to assume our exploratory findings with some caution.

## 5. Conclusion

We have confirmed the well-known association between HLA-C∗06 and type I psoriatic disease. As a novelty, our HLA-C∗06-positive patients appear to have a better cardiometabolic profile. Therefore, our findings could contribute to improving the overall care of these patients, paying special attention to the cardiometabolic health of HLA-C∗06-negative patients, and helping to a better selection of treatments for them. In that sense, a recent study reported that HLA-C∗06-negative patients are significantly more likely to respond to adalimumab than ustekinumab suggesting that reference to HLA-C∗06 status could offer substantial clinical benefit when selecting treatments for severe psoriasis [24].

## Figures and Tables

**Table 1:** Disease features of the study population.

Feature	*N*: 400
Mean age, years (SD)	46 ± 15
Mean age at psoriasis onset, years (SD).	29 ± 17.5
Mean time of disease evolution, years (SD)	17 ± 14.6
Men, *n* (%)	215 (53.8)
Women, *n* (%)	185 (46.2)
Mean PASI (SD)	12 ± 8.5
Psoriasis onset < 40, *n* (%)	280 (70)
Psoriasis onset ≥ 40, *n* (%)	120 (30)
Psoriasis family history, *n* (%)	168 (42)
PASI ≥ 10, *n* (%)	160 (40)
Nail involvement, *n* (%)	240 (60)
PsA, *n* (%)	93 (23.3)
Mean waist circumference, cm (SD)	97 ± 13.8
Mean BMI (SD)	28 ± 4.7
Hypertension, *n* (%)	80 (20)
Dyslipidemia, *n* (%)	64 (16)
Obesity, *n* (%)	120 (30)
Smokers, *n* (%)	72 (18)
Diabetes, *n* (%)	42 (10.5)
CHD, *n* (%)	25 (6.3)
Total cholesterol, mg/dl (SD)	205 ± 42.5
HDL-C, mg/dl (SD)	54 ± 14.9
LDL-C, mg/dl (SD)	128 ± 36.2
TG, mg/dl (SD)	126 ± 80.3
Blood glucose, mg/dl (SD)	101 ± 32.7
Topical/phototherapy treatment, *n* (%)	180 (45)
Nonbiologic systemic treatment, *n* (%)	248 (62%)
Biological therapy, *n* (%)	172 (43)

SD: standard deviation; *n*: number; PASI: psoriasis area and severity index; PsA: psoriatic arthritis; BMI: body mass index; CHD: coronary heart disease; HDL-C: high-density lipoprotein cholesterol; LDL-C: low-density lipoprotein cholesterol; TG: triglycerides.

**Table 2:** Distribution of variables between HLA-C∗06-positive versus negative subjects.

Variable	HLA-C∗06 positive *n*: 160	HLA-C∗06 negative *n*: 240	*p* values
Age (yrs), mean (SD)	43.3 (13.8)	48 (15)	<0.05^∗∗^
Age (yrs) at psoriasis onset, mean (SD)	22 (14.8)	32.6 (17.6)	<0.05^∗∗^
Disease duration (yrs), mean (SD)	19.2 (14.3)	15.3 (14.7)	NS
Men, *n* (%)	88 (55)	127 (53)	NS
Women, *n* (%)	72 (45)	113 (47)	NS
PsA, *n* (%)	34 (21.3)	59 (24.6)	NS
Psoriasis family history, *n* (%)	88 (55)	80 (33.3)	<0.05^∗∗∗^
PASI ≥ 10, *n* (%)^∗^	80 (50)	80 (33.3)	<0.05^∗∗∗^
Total cholesterol, mean (SD)	203.2 (41.8)	206.7 (43.4)	NS
HDL-C, mean (SD)	54.1 (15.4)	53.7 (14.8)	NS
LDL-C, mean (SD)	125.6 (36.7)	129.5 (36)	NS
TG, mean (SD)	125.1 (75.9)	128.7 (86.5)	NS
Glucose, mean (SD)	97.5 (32.6)	103.8 (33.9)	NS
BMI, mean (SD)	27 (4.4)	28.2 (4.8)	<0.05^∗∗^
Waist perimeter (cm), mean (SD)	94.4 (13.7)	98.3 (13.8)	<0.05^∗∗^
Hypertension, *n* (%)	22 (13.8)	58 (24.2)	<0.05^∗∗∗∗^
Dyslipidemia, *n* (%)	21 (13.1)	43 (17.9)	NS
Obesity, *n* (%)	42 (26.3)	78 (32.5)	NS
Smoker, *n* (%)	32 (20)	40 (16.7)	NS
CHD, *n* (%)	8 (5)	17 (7.1)	NS
Topical/photo therapy, *n* (%)	48 (30)	132 (55)	<0.05^∗∗∗^
Systemic treatment, *n* (%)	88 (55)	160 (66.7)	NS
Biologics, *n* (%)	90 (56.3)	82 (34.2)	<0.05

Yrs: years; SD: standard deviation; *n*: number; PsA: psoriatic arthritis; PASI: psoriasis area and severity index; BMI: body mass index; CHD: coronary heart disease; HDL-C (mg/dl): high-density lipoprotein cholesterol; LDL-C (mg/dl): low-density lipoprotein cholesterol; TG (mg/dl): triglycerides. ^∗^These values refer to the first visit to the clinic; ^∗∗^Student's *t*-test; ^∗∗∗^chi-square test; ^∗∗∗∗^Fisher's exact test.

## Data Availability

All data and additional information regarding this study are available to third parties under reasonable request. Patient's cohort was registered as a Biobank Collection by Spanish Instituto de Salud Carlos III (reference C.0003441).
